# Can we improve the management of inoperable malignant bowel obstruction? Results of a feasibility study of elemental diet as an alternative to parenteral nutrition in patients with advanced gynaecological cancer

**DOI:** 10.1007/s00520-024-08709-7

**Published:** 2024-08-02

**Authors:** Lindsey L. Allan, Simon S. Skene, Kate Bennett Eastley, Rebecca Herbertson, Eleanor Smith, Agnieszka Michael

**Affiliations:** 1 Royal Surrey NHS Foundation Trust, Egerton Road, Guildford, GU2 7XX United Kingdom; 2 Surrey Clinical Trials Unit, School of Biosciences, University of Surrey, Egerton Road Guildford, GU2 7XP United Kingdom; 3 University Hospitals Sussex NHS Foundation Trust, Royal Sussex Hospital, Eastern Road, Brighton, BN2 5BE United Kingdom

**Keywords:** Inoperable bowel obstruction, Ovarian cancer, Parenteral nutrition, Elemental diet

## Abstract

**Purpose:**

Nutrition support in inoperable bowel obstruction (IBO) remains challenging. Parenteral nutrition (PN) is recommended if the prognosis is > 2 months. An elemental diet (ED) is licensed for strictures in Crohn’s disease but has not been used in malignant bowel obstruction. The aim of this study was to evaluate the use of ED in patients with IBO and provide a proof of concept of ED as an acceptable feeding option.

**Methods:**

This was a mixed-methods single-arm feasibility study. The primary endpoint was to provide a ‘proof of concept’ of ED as an acceptable feeding option for patients with IBO. Secondary endpoints included taste acceptability, incidences of vomiting and pain, the proportion of women who tolerated ED, the number of cartons drunk, quality of life (QOL) and the number of women treated with chemotherapy. Patients (> 18 years) with CT-confirmed IBO who could tolerate 500 ml of liquid in 24 h remained on the trial for 2 weeks.

**Results:**

A total of 29 patients were recruited; of those, 19 contributed to the analysis for the primary endpoint; 13 (68.4%) participants tolerated the ED; 26 patients contributed to MSAS and EORTC QLQ questionnaires at baseline to allow for the assessment of symptoms. At the start of the study, 18 (69%) of patients experienced vomiting, reducing to 4 (25%) by the end of day 15 of the study; 24 (92%) of patients reported pain at consent, reducing to 12 (75%) by the end of day 15. QOL scores improved from 36.2 (95% CI 27.7–44.7) at baseline to 53.1 (95% CI 40.3–66) at the end of day 15; 16 (84%) participants commenced chemotherapy within the first week of starting ED. The number of cartons across all participants showed a median of 1.3 cartons per day (range 0.8 to 2.5).

**Conclusion:**

ED is well tolerated by patients with IBO caused by gynaecological malignancies and may have a positive effect on symptom burden and QOL.

## Introduction

Inoperable malignant bowel obstruction (IBO) is a common occurrence in patients with ovarian cancer. The rates reported in the literature are mainly based on ovarian and colorectal cancer complications and vary from 5 to 51% and 10 to 28%, respectively [1–3]. Epidemiological data in other malignancies is less well reported. Bowel obstruction is frequently the terminal event in the course of malignant disease, and few treatment options make a significant impact at this stage. Surgical interventions can be considered, but unfortunately, many patients present with bowel strictures caused by peritoneal carcinomatosis and are subsequently deemed inoperable [4]. This is due to the widespread nature of metastatic disease occurring at several sites within the small and large bowel mesentery, peritoneum and the surface of the bowel. This presentation is frequently coupled with multiple relapses, resistance to chemotherapy and therefore very poor prognosis. The survival rate for patients with cancer-related carcinomatous bowel obstruction (excluding the initial presentation when the disease is still sensitive to chemotherapy) is limited to a few weeks [5–7].

Patients frequently present with nausea and vomiting, which can be controlled with anti-emetic drugs, anti-secretory drugs and/or insertion of a nasogastric tube for drainage and to allow bowel rest. Nutrition is usually a major issue since, depending on the degree of small and/or large bowel obstruction, many patients cannot tolerate oral or enteral feeding (EN). This is particularly common in those with widespread peritoneal disease, causing recurrent symptoms after eating solid food. Due to the lack of well-designed clinical trials and inconsistent results, there is controversy regarding recommendations on the use of clinically assisted nutrition (EN or parenteral nutrition (PN)) in palliative care patients [8]. Current guidelines suggest that EN or PN should be considered in patients ‘with an inability to ingest or absorb sufficient nutrients’ [9–12]. In cancer patients, PN is recommended if malnutrition is more likely to cause progression than the disease itself [13–15]. Despite the recommendations, there is no consensus about the use of PN in patients with cancer, its effectiveness and the optimal timeframe for the introduction of feeding [16, 17]. The nutritional management of IBO varies according to local policy and often presents a major dilemma due to the variance in symptoms, as well as the location and severity of the disease.

Home PN is widely adopted in Europe and the USA but to a lesser extent in the UK. This may be due to the financial pressures of the NHS, as well as poor access to specialist units with appropriate MDT teams trained to provide PN in the community [18]. Reviews of the literature have shown that home PN is expensive and demonstrated that there is insufficient evidence to show that it positively impacts patient’s quality of life [19, 20]. One in four patients registered for home PN in the UK is diagnosed with a malignancy. The number of gastrointestinal obstructions has increased more than any other condition, with ovarian cancer being the most common reason [21]. The low number of patients in the UK having PN at home, as well as few successful surgical interventions undertaken at a late stage of disease, indicate that the vast majority of patients in the terminal phase of their illness with IBO may not be offered any nutrition and are discharged home without it [21]. This is potentially due to a lack of well-researched protocols on how to manage feeding in patients at the end of life.

### Elemental diet

An elemental diet (ED) is a liquid diet that contains proteins in the form of amino acids and fats in the form of medium-chain triglycerides, vitamins and trace minerals. EDs are almost completely absorbed in the upper small intestine, leaving only the endogenous residue to enter the large bowel [22]. ED is completely water-soluble, and therefore, it can be administered when solid foods are not tolerated. Standard oral nutritional supplements offered to patients with weight loss and poor appetite provide a polymeric diet that contains whole proteins requiring normal bowel digestion, which may not be tolerated by patients with IBO. Current indications for ED are intractable malabsorption, short bowel syndrome, inflammatory bowel disease and bowel fistulae [23]. ED in bowel obstruction has been successfully used and reported in one case report [24].

There are several studies evaluating ED in Crohn’s disease and comparing it to a polymeric diet or drugs. These show that it is accepted and that using liquid ED allows for bowel rest [25, 26] and may aid the healing to a level comparable to drugs such as steroids [27].

The current standard of care for patients with IBO in the UK is to admit them to a hospital and manage the IBO under a multi-disciplinary team’s care [12, 28]. Patients for whom further treatment is not deemed appropriate, such as surgery, chemotherapy or home PN, are often discharged with community palliative care support. Management is focussed on symptom control such as nausea, abdominal pain and distention and optimising quality of life in the terminal phases of the disease [29, 30]. Due to a lack of research, guidance and consensus, there is less focus on nutritional interventions, which can be very distressing for the patients, their families and the treating medical team [31, 32]. This has a profound effect on quality of life. ED has not been researched in the management of IBO despite its benefits in other diseases, and the focus of studies looking a nutrition in IBO is centred around parenteral nutrition [33].

Given the evidence for the use of ED in inflammatory bowel disease and malabsorption, we have conducted a feasibility study aiming to evaluate ED as an acceptable feeding option in patients with IBO caused by gynaecological malignancies.

## Materials and methods

### Trial design and participants

This was a mixed-methods single-arm multi-centre feasibility study. The primary objective was to provide a ‘proof of concept’ of ED as an acceptable feeding option for patients with IBO. The primary endpoints were taste acceptability of ED and incidence of vomiting and pain. The diet was considered as tolerated if the participants could take any amount of ED on more than half the study days (> 7 days) recorded, without a break of > 48 h, according to the nutrition diary. Taste acceptability was assessed by a 1 to 5 scale ranging from ‘1: I really like the taste’ to ‘5: I do not like the taste and cannot drink it’, and the incidence of vomiting and the incidence of pain were assessed by Memorial Symptom Assessment Scale (MSAS) [34]. The secondary endpoints assessed the number (proportion) of women who tolerated ED following presentation with IBO and were subsequently treated with palliative chemotherapy (as per local guidelines), the number of patients alive at the end of the study, Health Related Quality of Life from the European Organization for Research and Treatment of Cancer Quality of Life Questionnaire (EORTC-QLQ-C30) [35] and number of ED cartons taken in 24 h.

Eligible patients had advanced metastatic gynaecological cancer, were over 18 years and had CT-confirmed IBO due to disseminated malignancy. All patients were reviewed by the surgical team, the CT was discussed by a multi-disciplinary team, and a decision was made on whether operative management of obstruction was an option. Inoperable patients were then assessed to determine whether they were able to tolerate at least 500 ml of water orally (without vomiting for 24 h) using fluid balance charts. All patients were assessed for capacity to consent. Following informed written consent, participants remained on the trial for 2 weeks.

All available flavours were offered to patients, and patients were allowed to choose which flavour they preferred (pineapple, orange, grapefruit and summer fruits).

Patients were excluded who were unable to tolerate 500 ml of liquid in 24 h without vomiting or experiencing increased abdominal pain and distention. Women who had palliative surgery for an obstruction (i.e. diverting colostomy/ileostomy) were excluded from the study. Patients were allowed to have anti-emetics before they started the study with ED.

### Treatment and study procedures

ED was provided in the form of Elemental 028 Extra Liquid (E028) (Nutricia). Each 250 ml carton contains 215 kcal and 6.3 g protein and is available in three flavours: grapefruit, orange and pineapple and summer berries. Individualised advice on the number of cartons to be drunk was given following intervention from a specialist oncology dietitian. Nutritional intake was monitored throughout the study in a study-designed nutrition diary. Participants were asked to complete this daily and provide details of the number of cartons of E028 consumed. They were also asked to comment on the taste acceptability of all flavours of E028 at the end of week 1 and week 2. Whilst nutritional intake of other liquids was collected in the diary, this was excluded from analysis since this was not one of the objectives of the study.

If patients experienced vomiting, the ED was only implemented once the symptoms were controlled with anti-emetics. Other symptoms, such as bloating, did not delay the implementation of the ED.

A small cohort of patients was approached for an interview and were asked questions regarding their perspectives on participating in the trial and the ED. Interviews were audio-recorded and transcribed verbatim to analyse the opinions and feedback on ED. There was no formal qualitative analysis or topic guides apart from the ED feedback.

### Efficacy and safety assessments

Patients were assessed on day 1, day 8 and day 15, as well as at the point of discharge from the hospital if admission occurred during the trial. Symptoms and quality of life data were collected at each review using the MSAS [34] and the EORTC-QLQ-C30 [35].

No data was collected beyond the end of the trial period, but all participants remained under the care of the dietitian. If a benefit from the intervention was perceived, participants were given ongoing dietetic support to continue with the ED. Those who did not tolerate or benefit from ED were provided with individualised nutrition advice and alternative nutritional supplements if this was deemed appropriate. Safety was assessed throughout the study, and patients were given access to the acute oncology hotline as per local practice. Events considered to be related to underlying cancer progression, such as accumulation of effusions, ascites, thromboembolic disease or death, and which were not symptoms of bowel obstruction (such as nausea, vomiting, abdominal distention and pain), were not considered adverse or serious adverse events in the context of the current study.

Chemotherapy treatment decisions were made by the oncologists as per local guidelines.

### Sample size and statistical considerations

As this was a feasibility study to evaluate whether ED was an acceptable intervention for patients with IBO, data from 25 evaluable patients was considered sufficient and would allow estimation of the true tolerability rate within a standard error of 10%. As the prognosis is poor in this cohort, we assumed a 25% attrition rate and targeted recruitment of 34 patients.

Since the number of women with IBO who can tolerate ED was unknown (due to the novelty of the approach), we assumed a 50–50 chance of tolerability, with a sample size of 25. We expected at least 10/25 responders with close to 90% probability (88.5%), and the lower bound of a 95% confidence interval would indicate that the true tolerability would be > 20%.

Ethical approval was obtained from the Southeast Coast – Surrey Research Ethics Committee (reference 16/LO/2079) in accordance with the Declaration of Helsinki. The trial was sponsored by Royal Surrey NHS Foundation Trust and was included on the National Institute for Health Research clinical trials portfolio for ovarian cancer. Funding was provided by Target Ovarian Cancer UK Charity, and statistical oversight was by the Clinical Trials Unit at the University of Surrey. E028 was provided by Nutricia.

## Results

### Trial conduct and patients’ characteristics

The trial started recruiting in April 2017 and was completed in March 2020; 29 women aged 35–91 (median age 75.5 years) with IBO caused by a gynaecological malignancy were recruited to the study (Table 1).

**Table 1:** Clinical characteristics of the study population (29 pts)

Clinical characteristics		*N*		%
Type of cancer	Ovarian/fallopian tube cancer	17		59
	Primary peritoneal cancer	6		20
	Endometrial cancer	2		7
	Pelvic malignancy origin unspecified	2		7
	Data missing	2		7
Histology	High-grade serous cancer	22		76
	Malignant mixed Mullerian tumour	1		3
	Endometroid carcinoma	0		0
	Low-grade serous cancer	1		3
	Unknown/unspecified	5		18
FIGO stage	I	1		3
	II	1		3
	III	15		52
	IV	6		21
	Unknown/unspecified	6		21

Twenty-three women (79%) had ovarian, fallopian tube or primary peritoneal malignancy, 2 (7%) had endometrial cancer, and 2 (7%) had a pelvic malignancy with uncertain primary site. Data regarding the origin of the primary site of pelvic malignancy was missing for two patients. Most patients had high-grade serous cancer – 22 (76%) and 21 (73%) were classified as FIGO stage III or IV (see Table 1 for clinical characteristics).

**Fig. 1:**
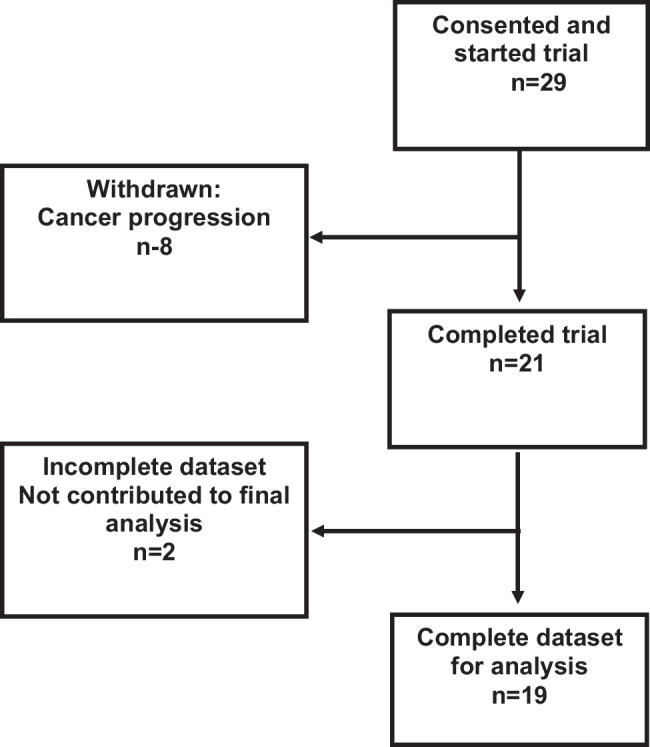
Breakdown of final recruitment and reasons for withdrawal from the trial

19 (65%) patients contributed data to primary endpoint analysis, 8 (27%) patients developed progressive symptoms due to rapidly progressing underlying malignancy and were withdrawn; data was incomplete for 2 patients, and they were deemed not evaluable. The breakdown of final recruitment numbers and patients who were withdrawn from the trial is summarised in Fig. 1.

### Efficacy

Thirteen out of the 19 evaluable participants (68.4%) tolerated the elemental diet as defined in the methods and, therefore, met the primary endpoint. The associated 95% continuity-corrected Wilson confidence interval for the proportion was 43.2 to 87.3%.

Nine patients (70%) of responders (those who tolerated ED) liked or found all three flavours (pineapple, orange, grapefruit and summer fruits) to be acceptable at the end of day 8. This increased to 10 patients (77%) of responders by day 15.

Twenty-six patients contributed to MSAS and EORTC QLQ questionnaires at baseline to allow for the assessment of symptoms, but the numbers reduced at week 1 and week 2, with only 16 patients contributing to week 2 MSAS and 15 to EORTC QLQ. The results are presented in Tables 2 and 3. ED did not appear to worsen nausea, vomiting, bloating or pain as measured by the MSAS questionnaire. These symptoms were experienced by fewer participants by the end of day 8 of the trial and were further reduced by the end of day 15. The severity of symptoms such as vomiting, pain and bloating all decreased by the end of day 8 and by the end of day 15 when none of the patients reported severe or very severe episodes of nausea (Table 2). At the start of the study, 18 (69%) patients experienced vomiting, reducing to 4 (25%) by the end of day 15 of the study; 24 (92%) patients reported pain at consent, reducing to 15 (75%) and 12 (75%) by the end of day 8 and day 15, respectively. In addition to vomiting and pain, patients also reported weight loss, which did not improve in the study (62% at the baseline and 63% at the end of the study) and bloating, which has improved (81% at baseline and 63% at the end of the study). All patients were receiving supportive care at the same time, including anti-nausea medication and painkillers; however, the intensity of the symptoms did not appear to worsen by continuing the study and ED.

**Table 2:** Results of the MSAS questionnaire

		Baseline	Week 1	Week 2
Number completing questionnaire		*n* = 26	*n* = 20	*n* = 16
		*n* (%)	*n* (%)	*n* (%)
Did vomiting occur?	Yes No Missing	18 (69) 7 (27) 1 (4)	5 (25) 15 (75) 0	4 (25) 12 (75) 0
How severe was it?	Slight Moderate Severe Very severe Missing	5 (28) 3 (17) 5 (28) 4 (22) 1 (6)	2 (40) 1 (20) 0 1 (20) 1 (20)	1 (25) 1 (25) 2 (50) 0 0
Was pain present?	Yes No	24 (92) 2 (8)	15 (75) 5 (25)	12 (75) 4 (25)
How severe was it?	Slight Moderate Severe Very severe Missing	6 (25) 7 (29) 5 (21) 5 (21) 1 (4)	4 (27) 4 (27) 3 (20) 2 (13) 2 (13)	4 (33) 5 (42) 2 (17) 0 1 (8)
Did bloating occur?	Yes No	21 (81) 5 (19)	14 (70) 6 (30)	9 (56) 7 (44)
How severe was it?	Slight Moderate Severe Very severe Missing	0 6 (29) 6 (29) 7 (33) 2 (10)	3 (21) 8 (57) 1 (7) 2 (14) 0	2 (22) 4 (44) 1 (11) 1 (11) 1 (11)
Did nausea occur?	Yes No Missing	21 (81) 5 (19) 0	8 (40) 11 (55) 1 (5)	8 (50) 8 (50) 0
How severe was it?	Slight Moderate Severe Very severe Missing	7 (33) 2 (10) 7 (33) 3 (14) 2 (10)	3 (38) 2 (25) 2 (25) 0 1 (13)	3 (38) 3 (38) 0 0 2 (25)

**Table 3:** EORTC QLQC30 nausea and vomiting scores (normalised scores)

Timepoint	*n*	Mean (SD)	Median (IQR)
Baseline	26	50 (35)	50 (33–67)
Week 1	20	16 (18)	17 (0–33)
Week 2	15	18 (20)	17 (0–33)

**Fig. 2:**
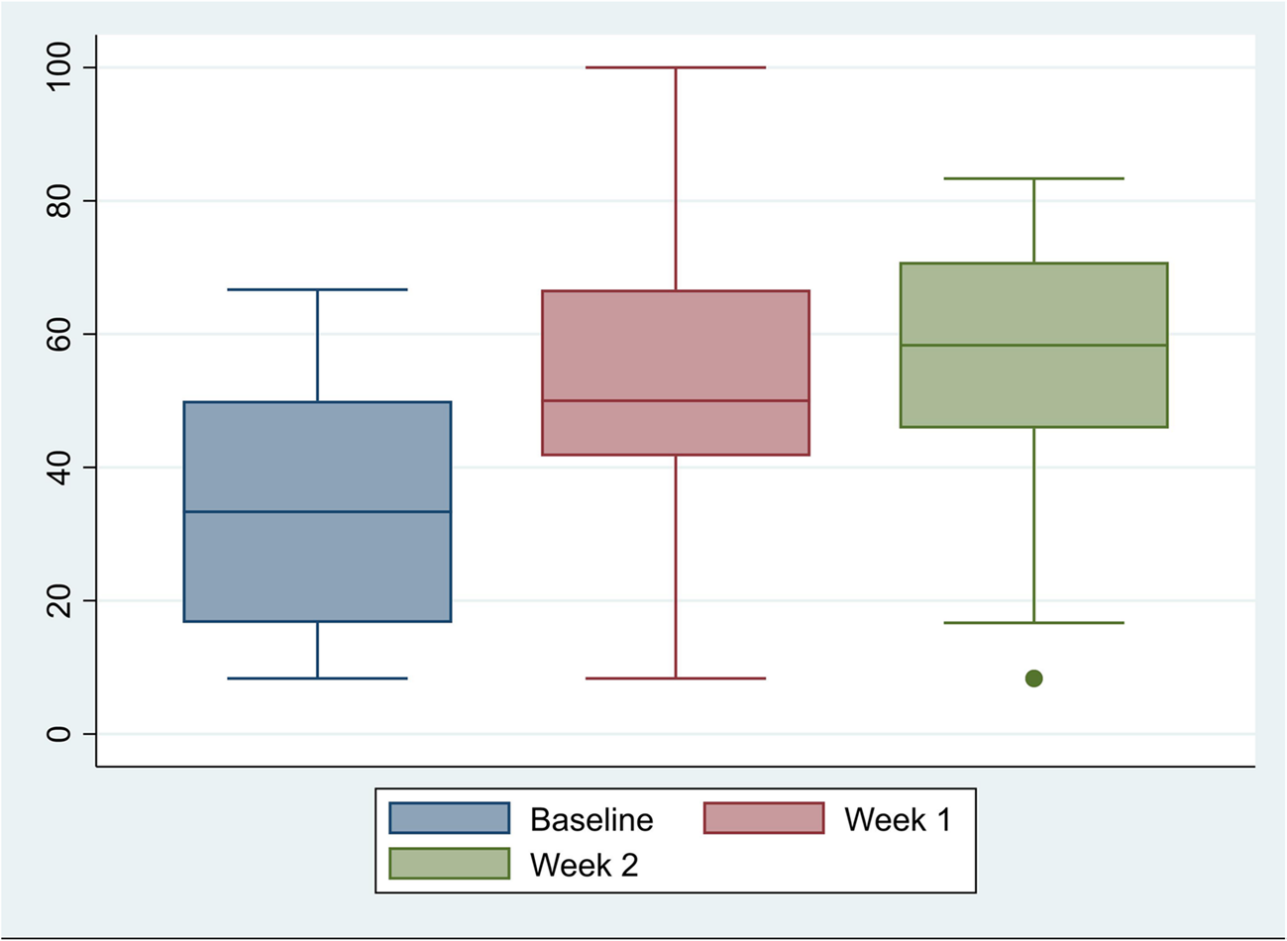
Changes in overall global health status QLQ-C30 scores

EORTC-QLQ-C30 QOL scores improved from 36.2 at baseline to 53.2 and 53.1 at the end of days 8 and 15, respectively. The differences between scores at baseline and subsequent weeks did not reach statistical significance (day 8: *p* = 0.015, day 15: *p* = 0.032) (Fig. 2).

### Chemotherapy

Of the 19 women evaluated for the primary endpoint, only 16 (84%) received chemotherapy either at the time of their first trial visit or started chemotherapy during the two-week trial period. In addition, two other women were reported as having chemotherapy planned, with an associated start date; 13 patients (68%) were able to start chemotherapy within the first week of starting ED as part of the study. Six of those had carboplatin alone, six had weekly paclitaxel, one had cisplatin, and two patients managed the combination of carboplatin and paclitaxel. One patient started 2 weeks of ED on gemcitabine and cisplatin.

### Nutritional intake

**Fig. 3:**
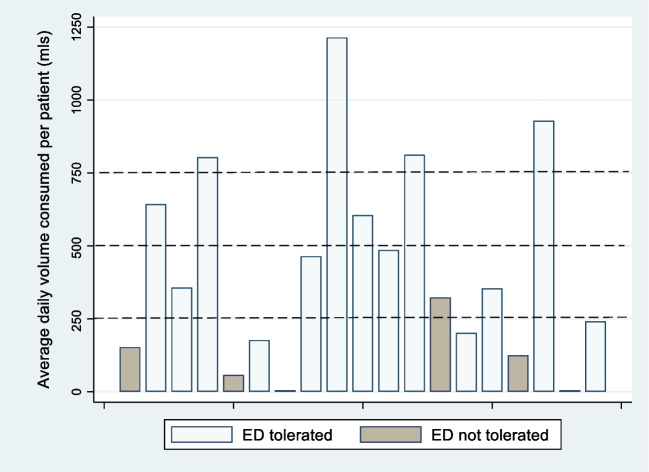
Number of daily cartons of ED taken by each participant

Nutritional intake of ED varied between each participant. The number of cartons across all participants showed a median of 1.3 cartons per day (range 0.8 to 2.5). The mean number of daily cartons taken by each participant is shown in Fig. 3. Data on other nutritional intake was not collected during the trial.

### Patients’ perspective

Four patients participated in semi-structured interviews and gave their views on ED and the trial. All felt that ED should be offered to patients with IBO, and none felt that it exacerbated their symptoms of obstruction. One participant said that ED improved her nutritional intake; ‘it helped me to give me extra fluids, definitely…so it was getting nourishment down’. Another commented that the ED ‘has meant that I’ve been able to cope with weekly chemo… so I’ve been really pleased about that’; ‘I’ve been able to continue to survive which is what I hoped would happen… I feel that the elemental that I’ve been able to stomach is doing its job.’ One participant implied it was an acceptable feeding option and had a positive impact on her quality of life; she later commented, ‘I’m glad that people are looking at something decent to help bowel obstruction cos it’s pretty grim, dismal thing to be in’. Only patients who were improving and felt better were approached to give their views, and therefore, the positive feedback may be biased.

## Discussion

IBO in advanced cancer has been shown to lead to increased symptoms of abdominal pain, bloating, nausea and vomiting, which have been widely reported in the literature [1, 3, 24]. The results of this study support these findings with over 80% of participants reporting bloating, nausea and pain at baseline, with 72% reporting nausea. These symptoms can be exacerbated by food and fluid intake in widespread peritoneal disease, potentially resulting in distress and reduced quality of life for patients [36]. The results of this feasibility study suggest that cancer patients diagnosed with IBO, who can tolerate some fluids orally, may be able to take ED as a form of nutrition and that drinking it is unlikely to lead to the deterioration in symptoms of bowel obstruction. Patients feel the ED helps them to obtain nutrition, and even in a critical situation, there is a perception that it gives them some strength and allows them to continue palliative treatment without the need to be admitted to the hospital.

Much of the research conducted on IBO has focussed on options for symptom control in complete bowel obstruction, with recommendations for the management of vomiting and abdominal pain, advice on placing venting gastrostomy tubes, and whether to commence PN as a feeding option [29, 30, 37]. There do not appear to be any published clinical trials on managing nutrition and diet in patients who have sub-acute obstruction and are able to tolerate some food and fluids. Meeting nutritional requirements orally in this patient group is challenging, if not impossible, and the emphasis should be placed on managing patient and carer expectations by ensuring that patients are not simply told to drink sips of water if PN is not an option and resolution of the obstruction is unlikely, i.e. in the case of those who are inoperable and have exhausted all treatment options. Qualitative research has shown that patients and family members place a lot of importance on food and fluids and the role of nutrition at this point [38]. Many have expressed concerns over what type of foods they can eat and struggle with the concept of no nutritional intake at all. A study by Sowerbutts et al. showed that patients experience a profound sense of loss at the inability of being able to consume food and fluids orally [39]. One case report described the anxiety of a patient who believed that she would starve to death before she passed away from her cancer diagnosis [40]. The comments made by the interviewed participants in this study support these views. The full qualitative study was not conducted, but some of the discussions with the patients indicate that a qualitative assessment examining the views of patients, their families and carers would be useful to further understand their attitudes and opinions.

A Japanese randomised controlled trial of Crohn’s disease patients assigned to either half-ED or normal diet [41] did not show any difference between quality of life scores using the Inflammatory Bowel Disease Questionnaire [42]. It is not possible to extrapolate the data to advanced cancer patients, and more research is required to investigate whether ED is a viable option in the palliative setting. The limitations of this study, the use of concomitant medication and other confounding factors related to an underlying malignancy do not make it possible to conclusively state that ED improves global health and quality of life. However, the changes in scores suggest that ED is well tolerated and should be considered as an alternative in patients for whom PN is not indicated and who are reaching the terminal stage of the disease. Only 26 women contributed to symptoms and quality of life assessment, and this is also a limitation of the study.

This study is the first to report the use of oral nutrition (in the form of ED) in patients with IBO. It demonstrates that the addition of ED to standard pharmaceutical interventions, such as anti-emetics, may lead to improvement in QOL, but some of the improvements may be due to concomitant supportive medications, spontaneously resolving obstruction or the effect of palliative chemotherapy. Due to the small sample size, these confounding factors were acknowledged but not included in statistical calculations to remove bias. Intake of calories or protein was not included in the trial objectives, so it is not possible to establish if improvements in QOL were related to an increase in nutritional intake. Many patients struggle with ED due to the severity of underlying disease and symptoms associated with IBO; a long-term ED is therefore uncertain, and further studies are required.

These results demonstrate that patients may be able to tolerate more than sips of water, which is usually advised in terminal cases and when PN is not appropriate. They complement existing guidelines that predominantly focus on the medical and surgical management of IBO and that have limited evidence to support nutritional recommendations [30, 43]. The findings may provide a means for reducing anxiety and distress reported by patients and carers in published qualitative studies.

## Conclusion

ED does not exacerbate symptoms and may improve QOL. More research is required to establish if it can be used as a nutrition option for IBO patients who can tolerate some fluids orally.

## Data Availability

Anonymised data avaialbe at request.
